# Complete Genome Sequencing and Comparative Genome Characterization of *Lactobacillus johnsonii* ZLJ010, a Potential Probiotic With Health-Promoting Properties

**DOI:** 10.3389/fgene.2019.00812

**Published:** 2019-09-10

**Authors:** Wei Zhang, Jing Wang, Dongyan Zhang, Hui Liu, Sixin Wang, Yamin Wang, Haifeng Ji

**Affiliations:** Institute of Animal Husbandry and Veterinary Medicine, Beijing Academy of Agriculture and Forestry Sciences, Beijing, China

**Keywords:** Lactobacillus johnsonii, complete genome sequence, comparative genome analysis, phylogenomic analysis, probiotic

## Abstract

*Lactobacillus johnsonii* ZLJ010 is a probiotic strain isolated from the feces of a healthy sow and has putative health-promoting properties. To determine the molecular basis underlying the probiotic potential of ZLJ010 and the genes involved in the same, complete genome sequencing and comparative genome analysis with *L. johnsonii* ZLJ010 were performed. The ZLJ010 genome was found to contain a single circular chromosome of 1,999,879 bp with a guanine–cytosine (GC) content of 34.91% and encoded 18 ribosomal RNA (rRNA) genes and 77 transfer RNA (tRNA) genes. From among the 1,959 protein coding sequences (CDSs), genes known to confer probiotic properties were identified, including genes related to stress adaptation, biosynthesis, metabolism, transport of amino acid, secretion, and the defense machinery. ZLJ010 lacked complete or partial biosynthetic pathways for amino acids but was predicted to compensate for this with an enhanced transport system and some unique amino acid permeases and peptidases that allow it to acquire amino acids and other precursors exogenously. The comparative genomic analysis of *L. johnsonii* ZLP001 and seven other available *L. johnsonii* strains, including *L. johnsonii* NCC533, FI9785, DPC6026, N6.2, BS15, UMNLJ22, and PF01, revealed 2,732 pan-genome orthologous gene clusters and 1,324 core-genome orthologous gene clusters. Phylogenomic analysis based on 1,288 single copy genes showed that ZLJ010 had a closer relationship with the BS15 from yogurt and DPC6026 from the porcine intestinal tract but was located on a relatively standalone branch. The number of clusters of unique, strain-specific genes ranged from 42 to 185. A total of 219 unique genes present in the genome of *L. johnsonii* ZLJ010 primarily encoded proteins that are putatively involved in replication, recombination and repair, defense mechanisms, transcription, amino acid transport and metabolism, and carbohydrate transport and metabolism. Two unique prophages were predicted in the ZLJ010 genome. The present study helps us understand the ability of *L. johnsonii* ZLJ010 to better adapt to the gut environment and also its probiotic functionalities.

## Introduction

The gastrointestinal tract (GIT) is home to a massive and diverse collection of symbiotic microbes. The GIT microbiota is essential to host health, as it provides nutrients (such as vitamins and short-chain fatty acids) and energy for the host *via* the fermentation of nondigestible dietary components. The GIT microbiota also plays a beneficial role in maintaining normal homeostasis by regulating the host’s immune system as well as influencing the development and physiology of tissues (Banerjee et al., 2018). Establishing and maintaining mutualistic host-microbe interactions are key requirements for host health. Interest in the beneficial functions of the gut microbiota has driven the identification of specific probiotic species with putative health-promoting capacities. The probiotic approach is also one of the alternatives to chemical and antibiotic treatment in the clinic, animal husbandry, and aquaculture.

*Lactobacillus johnsonii*, a member of the acidophilus group of the lactic acid bacteria, is often isolated from the intestines of humans and animals; and its beneficial properties, including exclusion or inhibition of pathogens (La Ragione et al., 2004), modulation of both local and systemic immune responses (Kingma et al., 2011; Lau et al., 2011), and enhancement of epithelial barrier function (Liu et al., 2015), have been extensively reported. As a result, *L. johnsonii* has been marketed as probiotic foods for humans and is also used to improve growth performance and prevent pathogen infections in the livestock industry. *L. johnsonii* L531 isolated from the colon of clinically healthy weaned piglets reduces pathogen load, helps maintain short-chain fatty acid levels, and maintains mucosal barrier integrity in the intestines of pigs challenged with *Salmonella enterica* Infantis, thereby controlling infection and improving growth performance (He et al., 2019; Liu et al., 2019). Prophylactic administration of *L. johnsonii* FI9785 leads to a reduction in *Campylobacter jejuni* colonization and maintains the gut microbiota homeostatis in chickens (Manes-Lazaro et al., 2017). Our previous study demonstrated that *L. johnsonii* ZLJ010, isolated from the feces of a healthy sow, represents a promising novel alternative to antibiotics for improving the reproductive performance and immune status of sows and restraining the growth of *Escherichia coli*, *Salmonella choleraesuis*, and *Staphylococcus aureus* (Wang et al., 2014). Dietary supplementation with ZLJ010 increases the immunoglobulin G level and decreases the alanine aminotransferase concentration in the serum of sows. ZLJ010 also increases the litter weight at birth and the weaning litter weight of piglets.

The wide range of functionalities of *L. johnsonii* strains suggests a high degree of variation in the content and type of their genomes. Analysis at the genome level is, thus, required to accurately understand and distinguish the characteristics of the strains of these microorganisms. Computational mining of sequences of genes, such as those involved in specific biosynthetic activities, or those encoding biofilm formation, may facilitate the selection and application of strains for specific biotechnological purpose. To date, the genomes of a number of *Lactobacillus* strains have been sequenced, such as *Lactobacillus brevis* (Feyereisen et al., 2019), *Lactobacillus plantarum* (Zhang et al., 2018), *Lactobacillus rhamnosus* (Jarocki et al., 2018), *Lactobacillus fermentum* (Illeghems et al., 2015), *Lactobacillus salivarius* (Harris et al., 2017), and *Lactobacillus gasseri* (Marcotte et al., 2017). Comparison of these sequences shows intra-species genomic rearrangements and significant genetic diversity among the *L. johnsonii* species, indicating towards a host-specific divergence of *L. johnsonii* strains with respect to genome inversion and phage exposure (Guinane et al., 2011).

To advance our understanding of the genome diversity and molecular evolution of *L. johnsonii* ZLJ010 strain, whole-genome sequencing of *L. johnsonii* ZLJ010 and genomic characterization were conducted. A comparative phylogenetic tree was constructed to reveal the evolutionary relationship between *L. johnsonii* ZLJ010 and other *Lactobacillus* strains. A comparative genome analysis was performed with seven other available complete genome sequences of *L. johnsonii* strains.

## Materials and Methods

### Bacterial Strain and Culture Conditions

*L. johnsonii* ZLJ010 was originally isolated from the feces of a healthy sow in our laboratory. The strain was identified through standard morphological, biochemical, and physiological tests, and by 16s rRNA gene sequencing using the nucleotide BLAST tool of the National Center for Biotechnology Information (NCBI). A single colony of *L. johnsonii* ZLJ010 was inoculated in modified De Man, Rogosa, and Sharpe (MRS) broth (20 g of sucrose, 20 g of soy peptone, 20 g of beef extract, 7.5 g of yeast extract, 5 g of sodium acetate, 2 g of diammonium hydrogen citrate, 2 g of potassium dihydrogen phosphate, 0.58 g of magnesium sulfate, 0.19 g of manganese sulfate, and 1 ml of Tween 80 *per* liter; pH 6.8) for 18 h at 37°C under microaerophilic condition. Stock cultures in modified MRS broth were mixed with 20% glycerol and stored at −80°C.

### DNA Extraction and Genome Sequencing

Bacterial cells were harvested by centrifugation, and total genomic DNA was extracted and purified using a Wizard Genomic DNA Purification Kit (Promega, Madison, WI) according to the manufacturer’s instructions. Purified genomic DNA was sheared into 8- to 10-kb fragments using G-tubes method, followed by preparation of SMRTbell libraries with C4 chemistry on the PacBio RS II System (Pacific Biosciences, Menlo Park, CA). Libraries were purified by removing short reads of <1.5 kb using 0.45× AMPure XP beads and quantified using Qubit Fluorometer (Thermo Fisher Scientific, Wilmington, DE). The sequencing primers were annealed to template DNA, and DNA polymerase enzyme C4 was added according to the manufacturer’s protocols. The enzyme/template complexes and libraries were loaded onto zero-mode waveguides using the DNA/Polymerase Binding Kit P6 (Pacific Biosciences). Single-molecule real-time (SMRT) cells were sequenced using the DNA Sequencing Reagent 2.0 Kit (Pacific Biosciences) with a 120-min sequence capture protocol and Stage Start to maximize the subread length on the PacBio RS II.

### Genome Assembly and Annotation

Raw sequence data were filtered to remove SMRTbell adapters, short polymerase reads of <100 bp and subreads of <1,000 bp, and low-quality reads of <80% accuracy using SMRT Analysis v2.3.0. A total of 84,207 reads with a median length of 2,921 bp were obtained for *de novo* assembly using the SOAPdenovo v2.04 (Luo et al., 2012). The data were subsequently analyzed on the Majorbio I-Sanger Cloud Platform (www.i-sanger.com). Genes were predicted using Glimmer v3.02 (www.cbcb.umd.edu/software/glimmer), and the corresponding function annotation was completed by blasting genes against Clusters of Orthologous Groups (COGs) of proteins and Kyoto Encyclopedia of Genes and Genomes (KEGG) databases. Tandem repeats were predicted using Tandem Repeat Finder v4.04, and the minisatellite DNA and microsatellite DNA were selected based on the number and length of repeat units. Further, rRNA, tRNA, and sRNA were predicted using rRNAmmer v1.2, tRNAscan v1.23, and Rfam v10.1, respectively. Genome visualization was performed using Circos v0.69-6. Putative prophage insert regions were detected using PHAST (http://phast.wishartlab.com/index.html), and the clustered regularly interspaced short palindromic repeats (CRISPR) were identified using MinCED 3 (https://sourceforge.net/projects/minced/). Transmembrane helices were identified using TMHMM Server v2.0. Signal peptide regions and their cleavage sites were predicted using SignalP Server v4.1. Carbohydrate-active enzymes (CAZymes) were searched against the CAZy database (http://www.cazy.org/). Antibiotic resistance genes were predicted using Comprehensive Antibiotic Resistance Database (CARD).

### Ortholog Clustering Analysis

A total of seven publicly available complete genome sequences of *L. johnsonii* strains were obtained from the NCBI database (**Table 1**). A list of the reference genomes is as follows: *L. johnsonii* NCC533 (NC_005362.1), FI9785 (NC_013504.1), DPC6026 (NC_017477.1), N6.2 (NC_022909.1), BS15 (CP016400.1), UMNLJ22 (CP021704.1), and PF01 (CP024781.1). The assembly levels of all genomes were “complete genome.” The orthologous gene set was built to identify the core-genome and pan-genome sizes using OrthoMCL package v2.0 (Li et al., 2003). All predicted protein sequences were merged together and compared with each other using BLASTP algorithm with *E*-value cutoff of 1e−5 and percent of match ≥50%. Then, all homologous protein pairs were parsed and grouped into orthologous families by cluster tool Markov Cluster (MCL), with an inflation value of 1.5. The core genome sizes were estimated by summing the orthologous families that contained genes from all the selected genomes. The pan genome sizes were estimated by summing all the orthologous families and the single genes together.

**Table 1:** Genome summary of *Lactobacillus johnsonii* strains.

Strain	Source	Size (Mb)	CDS	GC%	GenBank no.	Probiotic property	References
ZLJ010	Sow feces	2.00	1,959	34.9	CP032680	Inhibits pathogen growth and improves reproductive performance and immune status of sows	Wang et al., 2014
NCC 533	Human feces	1.99	1,821	34.6	NC_005362.1	Stimulates the innate immune response and increases Paneth cell number	Kingma et al., 2011
FI9785	Poultry	1.76	1,765	34.5	NC_013504.1	Competitive exclusion against pathogens	La Ragione et al., 2004
DPC 6026	Porcine gut	1.97	1,900	34.8	NC_017477.1	No reports	
N6.2	Rat	1.89	1,718	34.5	NC_022909.1	Inhibits type 1 diabetes *via* promoting Th17 differentiation	Lau et al., 2011
PF01	Piglet feces	1.93	1,846	34.6	CP024781.1	Inhibits the growth of *Escherichia coli* K88 and *Salmonella* spp.	Ahn et al., 2002
UMNLJ22	*Meleagris gallopavo* ileum	1.99	1,932	34.6	CP021704.1	No reports	
BS15	Yoghurt	2.11	1,944	34.9	CP016400.1	Promotes growth performance and lowers fat deposition by improving lipid metabolism and gut microflora in broilers	Wang et al., 2017

### Phylogenetic Analysis

The phylogenetic tree based on 16S rRNA gene sequences was constructed using the neighbor-joining method with MEGA software. The predicted amino acid sequences of each single copy orthologous gene family were aligned using MAFFT v7 (https://mafft.cbrc.jp/alignment/software/). The individual alignments were concatenated into a string of amino acid sequence alignment, and the concatenated alignment data were submitted to RAxML (https://github.com/stamatak/standard-RAxML) to build phylogenomic trees with the maximum-likelihood algorithm. The bootstrap method of 1,000 bootstrap repetitions was used to assess tree reliability.

### Strain Deposition and Complete Genome Sequence Data Accession Number

The sequence data for *L. johnsonii* ZLJ010 genome has been deposited at GenBank under the accession number CP032680. The strain has been deposited at the China General Microbiological Culture Collection Center (CGMCC no. 5762).

## Results and Discussion

### General Genome Features of *L. johnsonii* ZLJ010

**Figure 1:**
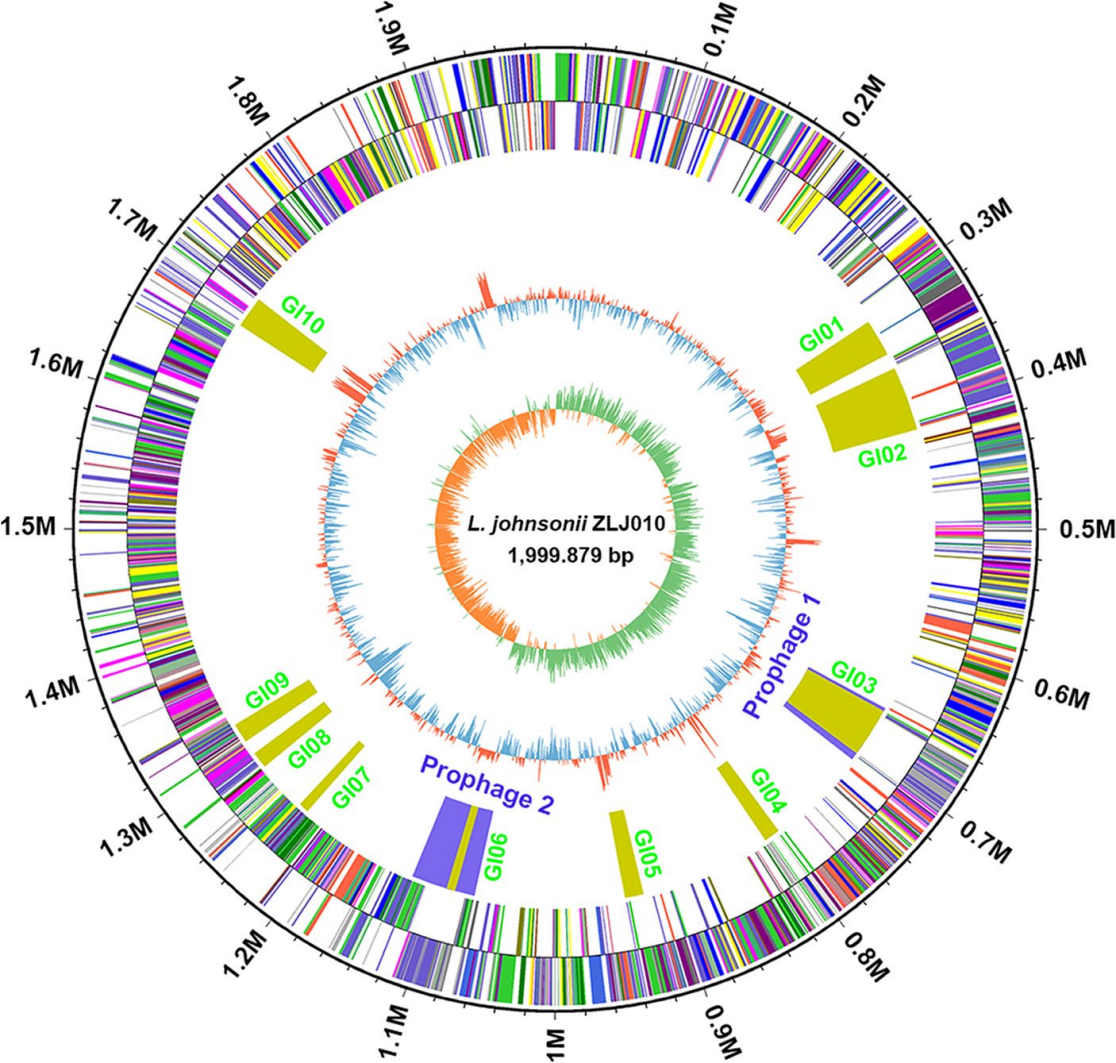
Circular genome map of *Lactobacillus johnsonii* ZLJ010. The circular map was generated using Circos and contain seven circles. Marked information is displayed from the outer circle to innermost, as follows: genome size, CDSs on the forward stand, CDSs on the reverse stand, prophage regions (blue) or genomic islands (GI, green), rRNA and tRNA, GC content and GC skew. CDSs, coding sequences; GC, guanine–cytosine; rRNA, ribosomal RNA; tRNA, transfer RNA.

The complete genome of *L. johnsonii* ZLJ010 contained a single circular chromosome of 1,999,879 bp with a guanine–cytosine (GC) content of 34.91% (**Figure 1**). No plasmids were present in the ZLJ010 genome. A total of 1,959 protein CDSs with an average length of 911 bp were identified, and these occupied 89.2% of the genome. Among the predicted CDSs, 1,474 genes (75.24%) were predicted as functional genes, and 485 genes (24.76%) were unknown and hypothetical genes. The chromosome contained 18 rRNAs, 35 tandem repeats, and 25 short interspersed repeats. Seventy-seven tRNA encoding sequences were identified, corresponding to all 20 natural amino acids: Leu and Met (7 sequences); Gly and Thr (6); Arg and Gln (5); Asn, Ile, Lys, Phe, and Ser (4); Asp, Glu, Pro, Tyr, and Val (3); Ala (2); Cys, His, and Trp (1); and an undermined amino acid (1). Compared with *L. brevis* (Feyereisen et al., 2019), *L. plantarum* (Zhang et al., 2018), *L. rhamnosus* (Jarocki et al., 2018), *L. fermentum* (Illeghems et al., 2015), *L. salivarius* (Harris et al., 2017), and *L. gasseri* (Marcotte et al., 2017), *L. johnsonii* had a smaller genome size and lower GC content, suggesting a high degree of variation in the genome and low genomic similarity among *L. johnsonii* strains to better adapt to the environment.

### Functional Classification

**Figure 2:**
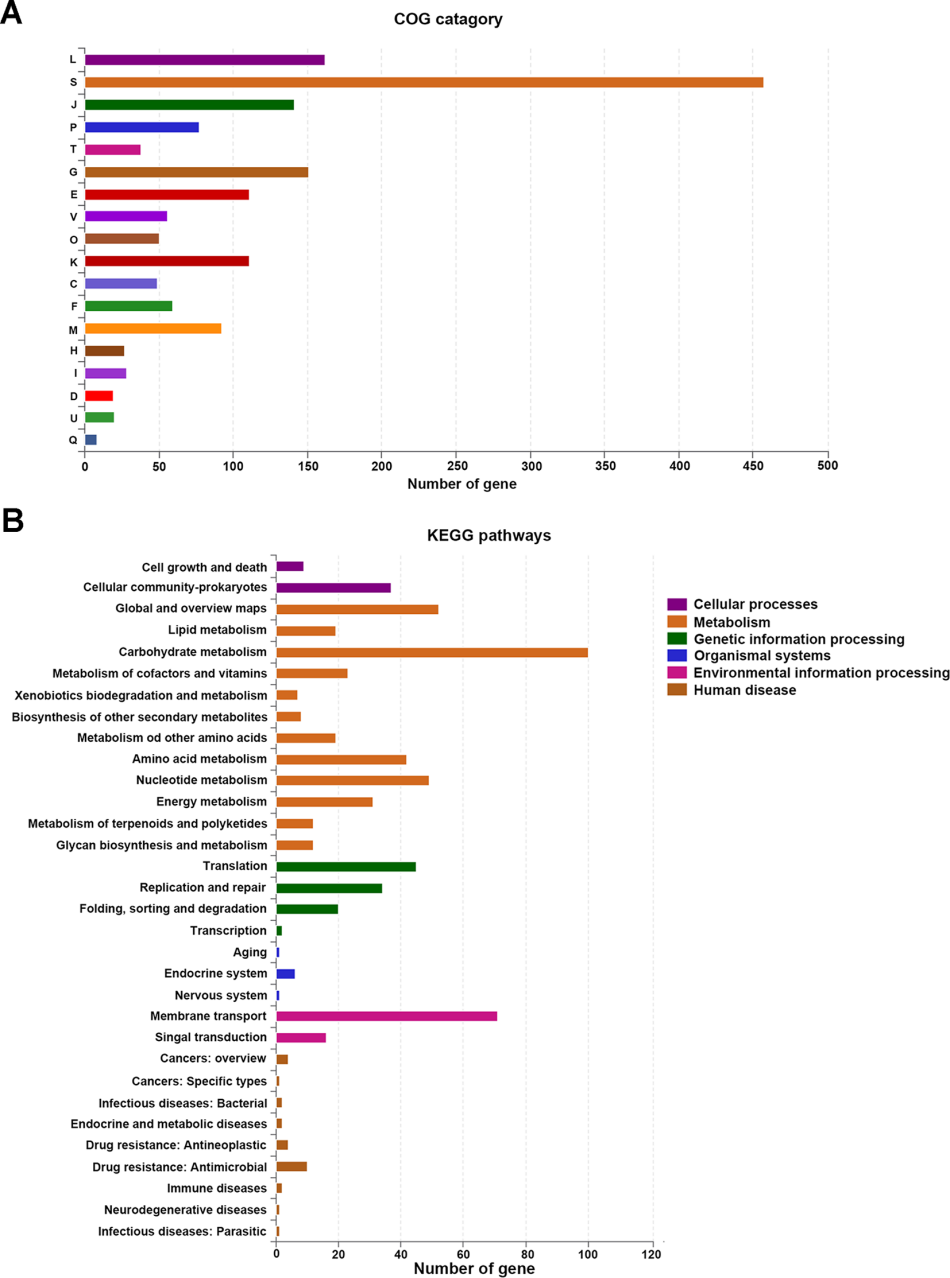
The number of genes assigned in COGs **(A)** and KEGG **(B)** categories. COGs, Clusters of Orthologous Groups; KEGG, Kyoto Encyclopedia of Genes and Genome.

Of the 1,959 CDSs, a total of 1,656 CDSs (84.53%) were specifically assigned to clusters of COG families comprising 18 functional categories (**Figure 2A**), among which function unknown (457 genes) was the most common term. In addition, other genes were mainly classified into function categories for replication, recombination and repair (162 genes); carbohydrate transport and metabolism (151 genes); translation, ribosomal structure, and biogenesis (141 genes); amino acid transport and metabolism (111 genes); transcription (111 genes); cell wall/membrane/envelope biogenesis (92 genes); inorganic ion transport and metabolism (77 genes); nucleotide transport and metabolism (59 genes); defense mechanisms (56 genes); posttranslational modification, protein turnover, and chaperones (50 genes); energy production and conversion (49 genes); signal transduction mechanisms (38 genes); lipid transport and metabolism (I, 28 genes); coenzyme transport and metabolism (H, 27 genes); intracellular trafficking, secretion, and vesicular transport (U, 20 genes); cell cycle control, cell division, and chromosome partitioning (D, 19 genes); and secondary metabolites biosynthesis, transport, and catabolism (Q, 8 genes). Furthermore, a total of 702 CDSs were classified to 32 KEGG functional categories and 151 functional pathways (**Figure 2B** and **Table S1**), mainly functioning in the biosynthesis of amino acids (ko: 01230; 64 genes), ribosome (ko: 03010; 54 genes), purine metabolism (ko: 00230; 53 genes), carbon metabolism (ko: 01200; 52 genes), ATP-binding cassette (ABC) transporters (ko: 02010; 45 genes), pyrimidine metabolism (ko: 00240; 40 genes), amino sugar and nucleotide sugar metabolism (ko: 00520; 33 genes), quorum sensing (ko: 0202 4; 31 genes), and fructose and mannose metabolism (ko: 00051; 30 genes). It has been proposed that gut-associated strains have adapted to their niche with a specialized set of metabolic and surface-related proteins (O’sullivan et al., 2009). Compared with *L. plantarum*, *L. johnsonii* strains have a relatively low number of genes involved in amino acid, carbohydrate and lipid metabolism, energy production and conversion, and transcription (Boekhorst et al., 2004).

### Phylogenetic Comparison of the *L. johnsonii* Strains

**Figure 3:**
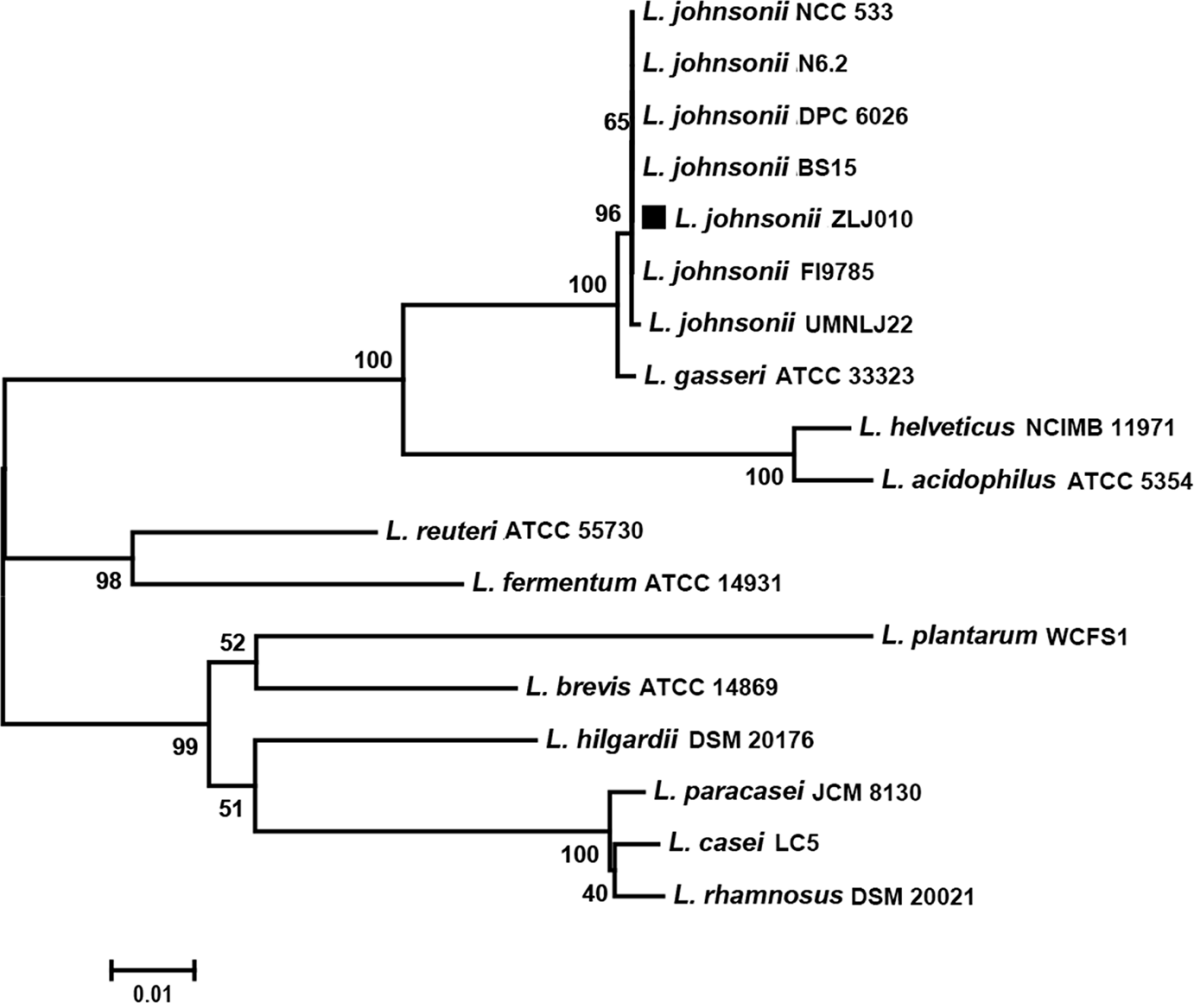
The neighbor-joining tree of *Lactobacillus johnsonii* ZLJ010 based on 16S rRNA gene sequence. The percent numbers at the nodes indicate the levels of bootstrap support based on neighbor-joining analyses of 1,000 replications. Bar, 0.01 nucleotide substitutions *per* site. rRNA, ribosomal RNA.

The phylogenetic tree based on the 16S rRNA gene sequences showed that the *L. johnsonii* members were grouped together but were distinguishable from other strains of the *Lactobacillus* genus. The strain ZLJ010 displayed a similarity of more than 99% to other *L. johnsonii* strains and showed a high degree of similarity with *L. gasseri* (**Figure 3**). Both *L. johnsonii* and *L. gasseri* are members of the closely related “acidophilus complex” of lactobacilli and are an autochthonous species of the gastro-intestinal tract (Fujisawa et al., 1992). The *L. johnsonii* genomes were difficult to distinguish by 16S rRNA gene sequence similarity. The availability of more complete genomes is required to demonstrate the composition of metagenomes and understand their evolutionary history in greater detail, beyond 16S rRNA gene analysis (Ellegaard and Engel, 2016).

**Figure 4:**
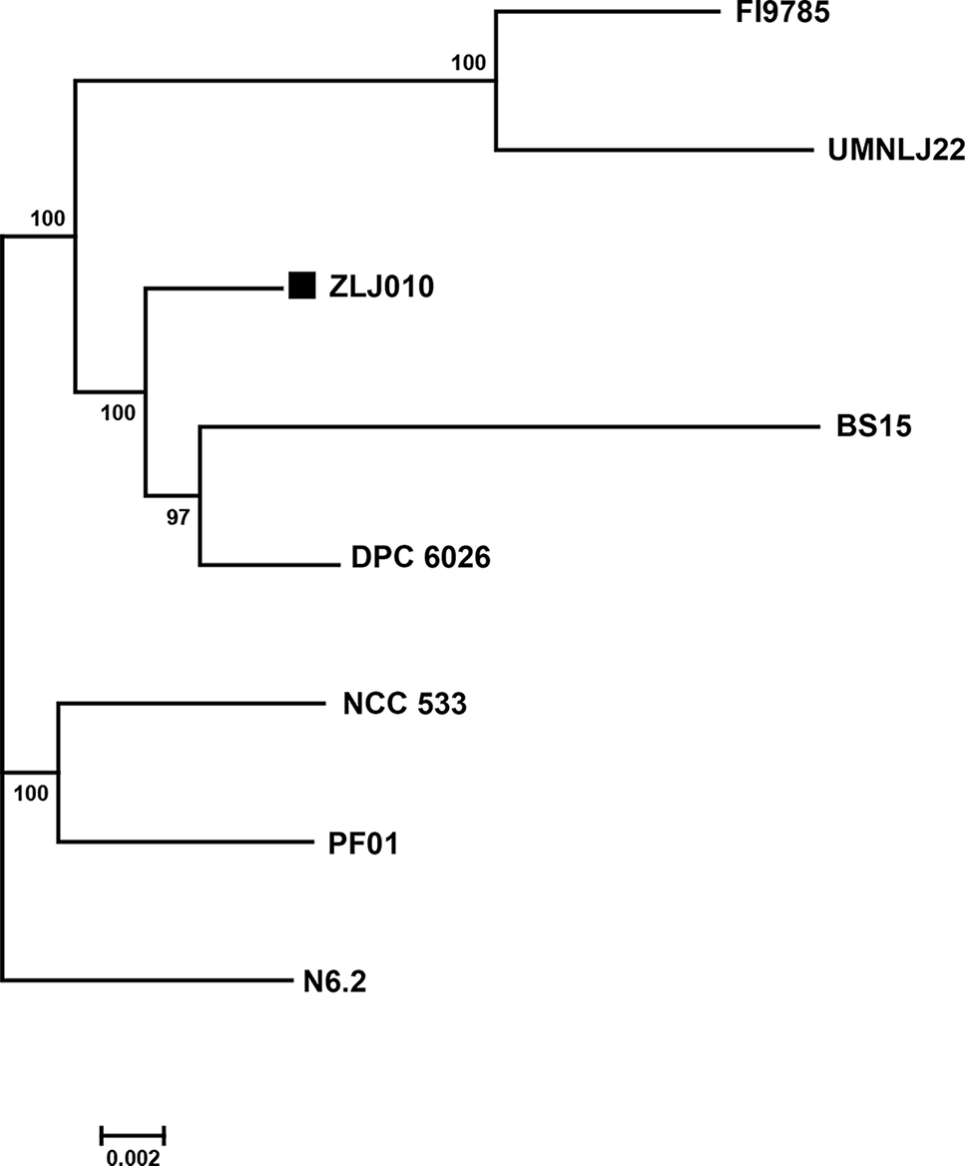
The phylogenetic tree of eight *Lactobacillus johnsonii* strains. The phylogenetic tree was built based on aligned concatenated sequences of single copy orthologous gene families. The bootstrap-support value before each node represents the confidence degree of each branch.

To further understand the phylogenetic relationship among the *L. johnsonii* strains, a phylogenetic tree was built based on 1,288 single copy genes of the orthologous gene clusters (**Figure 4**). The bootstrap value for each node was 100, except for one node (97) between BS15 and DPC6026. ZLJ010 showed a closer relationship with the BS15 and DPC6026 but was located on a relatively standalone branch. *L. johnsonii* BS15 is a probiotic strain that was first isolated from homemade yogurt. In broilers, *L. johnsonii* BS15 supplementation promotes growth performance and lowers fat deposition by improving lipid metabolism, intestinal development, and gut microflora (Wang et al., 2017). It also prevents subclinical necrotic enteritis by improving blood parameters related to immunity and enhancing intestinal immunity (Wang et al., 2018). *L. johnsonii* DPC6026 is a strain isolated from the porcine intestinal tract. The complete genome analysis revealed that *L. johnsonii* DPC6026 adapts to its niche by acquiring mobile genetic elements and through chromosomal recombination events (Guinane et al., 2011). It was demonstrated that grouping of the strains by origin does not occur, as shown by comparing the orthologous genes among the pangenome of 160 *L. plantarum* strains, and there is no potential link between the strain origin and genomic content (Jung et al., 2018). Our results indicated that the genome sequences may differ even in the same bacterial species, and phylogenomic analysis represents a reliable and high resolved approach to distinguish closely related species. *L. johnsonii* ZLJ010 has a distinguishing pattern of genomic evolution that allows it to adapt to the gut environment.

### Core and Pan Genomes of the *L. johnsonii* Strains

**Figure 5:**
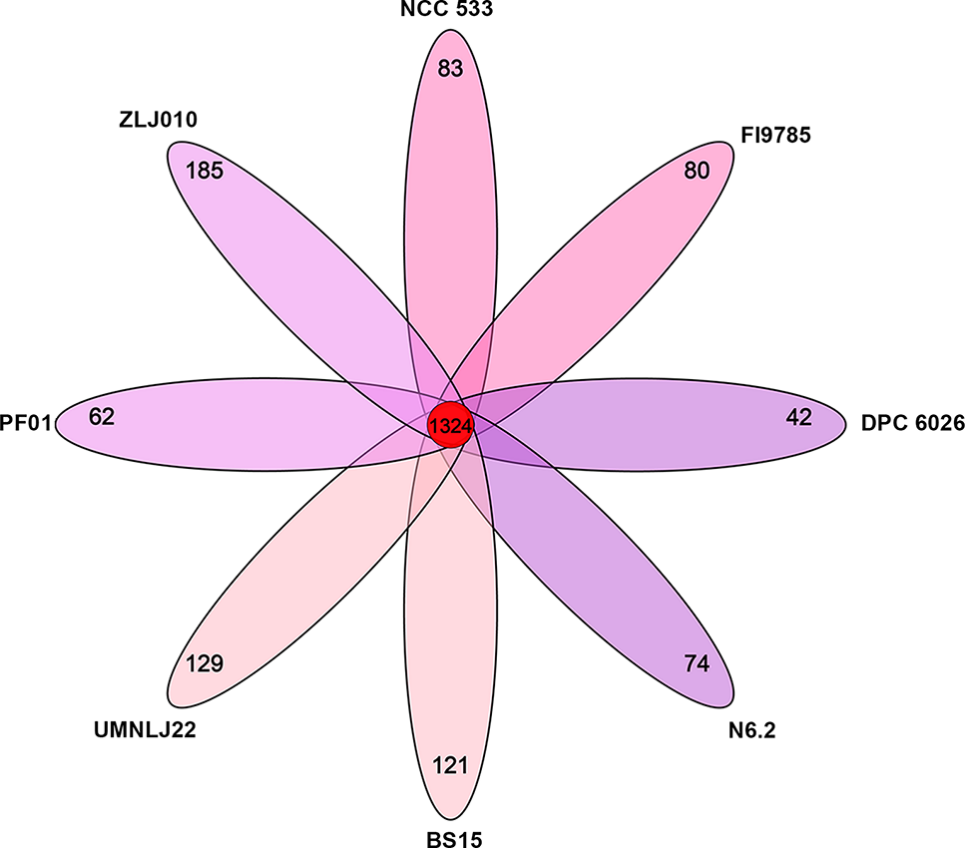
The numbers of orthologous gene families and unique genes among eight *Lactobacillus johnsonii* strains. The Venn diagram shows the number of orthologous gene clusters of the core genome (the center part) and the numbers of unique genes of each genome. The different colors indicate different sampling areas of the strains. The orthologous gene families were determined by OrthoMCL software with the inflation value of 1.5.

Core and pan genomes are usually used to evaluate genome diversity within a species or closely related bacteria. A core gene set of a species is the total gene numbers shared by all strains, which comprises genetic determinants to maintain the property of the species. Pan genome is a total gene pool reflecting the housing capacity of the genetic determinants. The OrthoMCL results showed that the genomes of *L. johnsonii* ZLJ010 and the other seven strain genomes had a pan genome of 2,732 orthologous gene clusters (**Figure 5**), which could be divided into a core genome of 1,324 orthologous gene clusters (48.46%) present in all eight strains, an accessory genome of 632 orthologous gene clusters (23.13%) present in a subset of the eight strains, and 776 strain-specific orthologous gene clusters (28.40%) (**Table S2**). The pan-genome size was 1.47 times the average size of these eight genomes, and the core genome constituted an average of 71.14% of each *L. johnsonii* genome. The formation of a large gene pool for those *L. johnsonii* members implied their open pan genome structures and, to a certain extent, endowed them with the capability of adaptation to the surrounding environments or their hosts. In the core genome, 1,173 gene clusters could be assigned in 19 COG categories (**Table 2**). The COG functional categories of some highly conserved biological processes, such as “translation, ribosomal structure and biogenesis” (Class J; 138 genes), “carbohydrate transport and metabolism” (Class G; 107 genes), “Secondary metabolites biosynthesis, transport and catabolism” (Class Q; 106 genes), “replication, recombination, and repair” (Class L; 87 genes), and “amino acid transport and metabolism” (Class E; 76 genes), were enriched among the core genome of *L. johnsonii*. Due to its smaller genome size, *L. johnsonii* has a higher percentage of genes related to “core functions” such as replication, translation, posttranslational modification, and cell cycle control (Boekhorst et al., 2004).

**Table 2:** The number of core orthologous gene clusters of *Lactobacillus johnsonii* and ZLJ010-specific 219 genes assigned in COG categories.

COG categories	Core orthologous gene cluster of *L. johnsonii*	ZLJ010-specific 219 genes
Unassigned	139	102
C	39	1
D	19	0
E	76	5
F	56	1
G	107	4
H	26	0
I	27	1
J	138	0
K	73	7
L	87	14
M	58	0
N	3	0
O	39	1
P	62	1
Q	106	0
S	272	73
T	29	1
U	17	0
V	25	8

COGs, Clusters of Orthologous Groups.

### Unique Genome Characteristics of ZLJ010

The number of strain-specific genes ranged from 42 gene clusters only found in DPC6026 to 185 gene clusters unique to ZLJ010. This result is in accordance with that of a previous study, which demonstrated 83–92% conserved genes between *L. johnsonii* isolates, with 5% strain-specific genes (Berger et al., 2007). ZLJ010 had 219 unique genes including 69 proteins with known functions and 150 hypothetical proteins of unknown function. Among the unique genes present in the ZLJ010 genome, 12 genes were involved in the ABC transporter system, and one gene was involved in the phosphotransferase system (PTS) transporting glucose. The remaining unique gene families encode proteins that could benefit the fitness of the strain such as prophages, or cell wall polysaccharide synthesis, such as genes predicted to encode lipoprotein.

To further investigate the functionality and diversity of proteins encoded by the unique genes, COG and KEGG analyses were performed. Of the 219 unique genes in the ZLJ010 genome (**Table 2** and **Table S3**), 117 genes (53.4%) were assigned in 12 COG functional categories. The COG class “unknown function” (Class S; 102 genes) was overrepresented among the unique genes, indicating that significant effort will be required to determine the putative functions carried out by the unique genes. In total, 14 genes were enriched in the “replication, recombination, and repair” (Class L). Among the eight genes assigned in “defense mechanisms” (Class V), two specific genes (gene0833 encoding the Mrr motif and gene1243 encoding the S subunit of restriction enzyme EC: 3.1.21.3) were assigned to the Type I Restriction Modification (R/M) system. R/M systems constitute one of the biological barriers exerted by a strain against foreign DNA (Dupuis et al., 2013). The defense mechanism category containing immunity proteins is specific for bacteriocin-producing strains and contributes to the resistance against bacteriocin (Diep et al., 2007). A total seven genes were assigned in “transcription” (Class K), containing several transcriptional regulator and antirepressors. These regulators act on specific genes to control their expression and confer an advantage when present in the gut. It would be interesting to determine the genes affected by these transcriptional regulators to assess the mechanisms employed to survive in this harsh environment. Five genes were assigned to “amino acid transport and metabolism” (Class E), and four genes were assigned to “carbohydrate transport and metabolism” (Class G). These unique genes may endow ZLJ010 with the ability to use extra energy and to defend itself against the adverse stimuli, so as to better adapt to the environment. For example, *L. johnsonii* ZLJ010 carried a distinguished glucose-specific IIB component (EC: 2.7.1.199) encoded by *ptsG* (gene1844). Due to the presence of the *bgaB* gene (gene0571) encoding beta-galactosidase (EC 3.2.1.23), beta-galactose may be utilized by ZLJ010 but not by the other *L. johnsonii* strains. Furthermore, among the 11 genes that could be classified into the KEGG functional pathways, six genes were involved functioned in quorum sensing (ko: 02024), including two *gadC* gene encoding the glutamate:γ-aminobutyric acid antiporter and four genes encoding ABC transporter binding proteins. GadC catalyzes the conversion of l-glutamate to γ-aminobutyric acid in a proton-consuming reaction, contributing to intracellular pH homeostasis and acid resistance (Ma et al., 2012). As mentioned above, a larger number of the unique genes are linked to replication, recombination and repair, and defense mechanisms. A strain in the gut would first need to resist other exogenous damage to maintain genomic integrity in order to survive the harsh gut environment.

### Lifestyle Adaptation to Stress

*L. johnsonii* ZLJ010 harbored many genes that code for stress-related proteins. The ZLJ010 genome contains the *Nhac* gene that codes for the Na^+^:H^+^ antiporter (gene1634) and the gene0895 that codes for choloylglycine hydrolase; these proteins are associated with tolerance to low pH and bile salts in the gut. ZLJ010 also carried various genes encoding heat stress proteins, including heat shock protein 20 (gene0190) and molecular chaperones Hsp33 (gene0295), GroEL (gene0447), DnaK (gene0876), and DnaJ (gene0877). Regulatory proteins are important for the adaptation of an organism to different environments. The ZLJ010 genome was found to encode a few involved in transcriptional regulation, including the sigma factor (gene0506, 0827, 0845, 0938, and 1687) and 85 transcriptional regulators. There were fewer regulatory proteins in the genome of *L. johnsonii* than *L. plantarum*. A relatively large number of regulatory proteins are present in the large genomes (Konstantinidis and Tiedje, 2004; van Nimwegen, 2003). The different lifestyles of *L. johnsonii* and *L. plantarum* also contribute to this difference. *L. plantarum* with its free-living lifestyle apparently has the regulatory machinery to adapt to different environmental niches, while *L. johnsonii* does not need a complex regulatory system for the relatively stable environment of the gut. The genes in the ZLJ010 genome may be involved in oxidative stress tolerance by coding for proteins such as thioredoxin reductase (gene1492), thiol peroxidase (gene1154), and glutaredoxin (gene1120) that catalyze glutathione-dependent disulfide reductions (Pophaly et al., 2012). ZLJ010 encoded genes encoding proteases involved in the stress response, such as the ATP-dependent intracellular proteases ClpP (gene1480) and HslV (gene1220), which prevent aberrant damage to proteins.

### Biosynthesis and Transport System

ZLJ010 lacked complete or partial biosynthetic pathways for amino acids. *L. johnsonii* is predicted to be incapable of synthesizing most, if not all, of the 20 standard amino acids. This reflects the environment that *L. johnsonii* inhabits—it is typically present only in the gut, although some reports indicated that *L. johnsonii* might also be present in other nutrient-rich environments (Guan le et al., 2003; Meroth et al., 2003), where it can take up peptides and amino acids from its environment. A considerable proportion of unique genes is linked to intracellular trafficking and vesicular transport in the *L. johnsonii* genome (Boekhorst et al., 2004). *L. johnsonii* has an extracellular, cell-bound proteinase to release these peptides from proteinaceous substrates and has more intracellular peptidases for the degradation of imported peptides than *L. plantarum* (Kleerebezem et al., 2003; Pridmore et al., 2004). However, ZLJ010 may synthesize Asn from Asp *via* aspartate-ammonia ligase (EC: 6.3.1.1) encoded by *asnA* (gene1945) and Gly from Ser *via* glycine hydroxymethyltransferase (EC: 2.1.2.1) encoded by *glyA* (gene0274). ZLJ010 may also synthesize Lys from Asp *via* a series of dehydrogenases, aminotransferases, reductases, deacetylases, and epimerases encoded by a complete gene cluster (gene1184–1192), which was absent in the NCC533 (Pridmore et al., 2004) and N6.2 (Leonard et al., 2014). ZLJ010 harbored the *glnA* gene (gene1546) encoding glutamine synthetase (EC: 6.3.1.2), indicating that ZLJ010 can interconvert Glu and Gln. Both *L. johnsonii* and *L. plantarum* can convert pyruvate to d- and l-lactate, but *L. johnsonii* lacks the pyruvate dehydrogenase and other enzymes necessary for the conversion of pyruvate to acetaldehyde, acetyl-coenzyme A, and acetate. *L. johnsonii* is an obligate homofermentative organism, capable of homolactic fermentation only, while *L. plantarum* is a facultative heterofermentative organism, capable of mixed-acid fermentation resulting in the formation of lactate, formate, and/or acetate depending on environmental conditions. The lack of the pyruvate dehydrogenase complex in *L. johnsonii* is consistent with the anaerobic environment in the gut. ZLJ010 had an enhanced transport system to compensate for its limited amino acid biosynthetic capabilities.

The *L. johnsonii* ZLJ010 genome carried 210 genes related to the transport system, mainly comprising the ABC transporter system and PTS (**Table S4**), suggesting exchange between the strain and its environment. The ZLJ010 genome harbored 170 genes encoding the ABC transporter system components. Many of these ABC importers transport peptides, amino acids, and inorganic ions; however, the substrate specificity of most of the exporters is unknown. The ZLJ010 genome carried four transporters for the uptake of branched-chain amino acids and 25 amino acid-permease type transporters, more than twice the number of transporters present in the majority of other lactic acid bacteria. A total of 11 genes involved in peptide transport were present in the ZLJ010 genome. A complete Glu-specific system (gene0579, gene0581–gene0583, and gene0596) was found in the ZLJ010 genome. ZLJ010 could acquire exogenous amino acids to fuel protein synthesis *via* these genes encoding specific ABC transporters.

Forty genes were related to the genomic PTS, and ptsI (gene1521) and gene1528 encoded PTS Enzyme I (EI) and phosphocarrier protein HPr, which delivered phosphoryl groups from phosphoenol-pyruvate to EII enzymes (EIIs) (Kotrba et al., 2001). The PTS is a major carbohydrate active-transport system in bacteria that catalyzes the phosphorylation of sugar substrates to cross the microbial cell membrane. The ZLJ010 genome encoded 11 complete phosphoenolpyruvate-dependent PTS EII complexes related to the transport of carbon sources, including alpha-glucosides, beta-glucosides, cellobiose, fructose, galactitol, glucose, lactose, mannose, sorbitol, sorbose, sucrose, and trehalose. These sugar PTSs can import more than one substrate, thereby expanding the carbon transport capacity of ZLJ010. *L. johnsonii* genome carried more PTS transporters than nearly all other microbes with similarly sized genomes. For example, ZLJ010 carried the genes for the specific transport of α-glucosides, lactose, sorbose, and trehalose, which cannot be utilized by *L. plantarum* ZLP001 (Zhang et al., 2018).

### Secretion and Extracellular Proteins

Components of the Sec-SRP secretion system were found in the ZLJ010 genome, including the signal-recognition particle proteins FtsY (gene0806) and Ffh (gene0836) and the component SecA (gene1498) as well as SecE (gene0394). There were also two membrane protein YidC homologs (gene1563 and gene2052) involved in the insertion of hydrophobic sequences into the lipid bilayer after initial recognition by the SecAYEG translocase (Scotti et al., 2000).

Extracellular proteins are important for the interaction of organisms with the surrounding environment, such as *via* communication and adhesion. This makes them of special interest in the case of lactobacilli, because they may be involved in interactions of microbe–microbe and host–microbe interactions, such as in the GIT or on plant materials. Putative extracellular proteins of ZLJ010 were identified based on the presence of a Sec pathway-dependent signal peptide; these comprise proteins that become attached to the cell surface and those that are secreted into the environment. The former were identified by searching for cell-anchoring domains, such as the C-terminal LPxTG motif and N-terminal lipoprotein motif for anchoring to peptidoglycan and the cell membrane, respectively. In total, 72 putative extracellular proteins with signal peptides were predicted in the ZLJ010 genome (**Table S5**), many of which might be related to amino acid transport (10 proteins, COG category: E), carbohydrate transport (5 proteins, COG category: G), inorganic ion transport (5 proteins, COG category: P), and cell wall/membrane/envelope biogenesis (4 proteins, COG category: M). Bacteria can synthesize cytoplasmic storage polysaccharides and exocellular polysaccharides (EPS) including the tightly linked capsular polysaccharides (CPS) and the loosely associated cell surface EPS (Zeidan et al., 2017). It was established that EPS are involved in the interaction of bacteria with their environment. CPS and EPS have been shown to be involved in the adhesion to abiotic and biotic surfaces (Caggianiello et al., 2016), formation of bacterial biofilms (Flemming and Wingender, 2010), and the interaction with the immune system (Laiño et al., 2016). ZLJ010 was predicted to encode a type 1 CPS biosynthesis protein (gene0985 and gene1662). EPS biosynthetic gene clusters from gene1309 to gene1322 encode five glycosyltransferases (gene1313–1317), two EPS biosynthetic enzymes (gene1320 and gene1322), and one UDP-galactopyranose mutase (gene1311). The flippase wzx (epsN) is also found in the EPS cluster of ZLJ010. A glucosamine-fructose-6-phosphate aminotransferase (GlmS) (E.C. 2.6.1.16, gene0735) was present in the genome of ZLJ010. GlmS is a rate-limiting enzyme in the biosynthesis of peptidoglycan, which forms the mesh-like layer in bacterial cell walls and induces the host innate immune response (Barreteau et al., 2008; Bron et al., 2012). ZLJ010 harbored gene involved in lipoteichoic acid (LTA) synthesis, including glycerol phosphate LTA synthase (gene1735) and d-alanyl-lipoteichoic acid biosynthesis protein (gene1986 and gene1988). LTA binds to membrane Toll-like receptor 2 and subsequently activates nuclear transcription factor kappa B involved in the innate immune response (Nguyen et al., 2017).

### Mobile Genetic Element Analysis

A total of 10 specific regions of genome plasticity containing 240 genes were detected in the ZLJ010 genome, designated here as genomic islands GI01–GI10, which were more than 0.2 Mb in size and contributed significantly to genome variability within this species (**Figure 1** and **Table S6**). One of the main differences of ZLJ010 from the other seven *L. johnsonii* genomes encompassed the presence of three GIs—GI03 (gene0641–0713), GI04 (gene0811–0827) and GI06 (genes1100–1108)—inserted in several parts of the genome. GI04 (13 kb) clearly did not contain phage-like genes but carried a gene (gene0811) encoding integrases of the tyrosine recombinase XerC family. These were located downstream of genes for tRNA^Gly^ (for GI4) and threonine-accepting tRNA (tRNA^Thr^ for GI04), suggesting the occurrence of site-specific insertion. The function of the other CDSs in GI04 was unknown.

PHAST predicted regions of GI03 and GI06 to be prophages (prophage 1 and prophage 2, respectively). Prophages are widely distributed in the genomes of *Lactobacillus* species, such as *L. salivarius*, *L. rhamnosus*, *Lactobacillus casei*, *Lactobacillus lactis*, *L. gasseri*, and *Lactobacillus kunkeei* (Ventura et al., 2006; Kankainen et al., 2009; Baugher et al., 2014). The prophages comprised approximately 5.07% of the ZLJ010 genome (**Figure 1** and **Table S7**). The prophage 1 region extended from 658,396 to 709,855 bp with a GC content of 36.23% and contained 81 CDSs with a complete prophage element from gene0638 to gene0721. The prophage 2 region extended from 1,069,282 to 1,124,377 bp with a GC content of 35.52% and contained 65 CDSs with a complete prophage element from gene1073 to gene1139. Integrases are useful markers for prophages in bacterial genomes. Two site-specific integrases (gene0641 and gene1075) were identified in the prophage 1 and 2 regions, respectively. Genes in the GI3 were homologous to genes of the bacteriophage B054 (NC_009813, prophage 1 region) found in *Listeria monocytogenes*. The prophage B054, classified into the Myoviridae family, features a contractile tail and can be induced by ultraviolet radiation (Dorscht et al., 2009). The GI06 region was the part of the prophage Lj928 (NC_005354, prophage 2 region) found in *Lactobacillus* (Ventura et al., 2003). As the 7-kb GI6 lies within the prophage 2 region (NC_005354, Lj928), it may have an evolutionarily distinct origin and may have been inserted into the phage-like region. Two complete prophages, Lj928 and Lj965, were detected in the genome of the NCC533 strain. The Lj928 prophage is restricted to NCC533-like strains sharing an essentially identical pulsed-field gel electrophoresis pattern (Ventura et al., 2003) and the deduced structural proteins from Lj928 demonstrated amid acid sequence identity with *Lactococcus lactis* phage TP901-1 (Ventura et al., 2004). Numerous phage elements may confer genome variations in the evolution of *L. johnsonii* ZLJ010.

CRISPR represent a family of DNA repeats providing acquired immunity against foreign genetic elements (Amitai and Sorek, 2016). They are typically composed of short and highly conserved repeats (∼30 bp), interspaced with variable sequences called spacers, and are often found adjacent to cas (CRISPR-associated) genes (Hille et al., 2018). CRISPR are found in approximately 40% of sequenced bacterial genomes (Sorek et al., 2008). The presence of CRISPR loci may increase the genome stability of a bacterial strain and, therefore, its adaptation in the environment. The *L. johnsonii* ZLJ010 genome contained five CRISPR loci (CRISPR1–CRISPR5) (**Table S8**). The detected CRISPR/CRISPR-associated (Cas) system was of type II-A (four cas genes; cas1, cas2, cas9, and csn2).

### CAZymes and Secondary Metabolic Product

The analysis of CAZymes revealed that the ZLJ010 genome contained 72 genes in the five CAZymes gene families (**Table S9**): 10 carbohydrate esterase (CE) genes, 3 carbohydrate-binding modules (CBMs), 28 glycosyl transferase (GT) genes, 28 glycoside hydrolase (GH) genes, 2 auxiliary activity (AA) genes, and 1 polysaccharide lyase (PL). GTs catalyze the transfer of sugars from activated donor molecules to specific acceptors and are important for the formation of surface structures, which are recognized by host immune systems (Mazmanian et al., 2008). The ZLJ010 genome carried a higher percentage of GT genes than *L. plantarum* ZLP010 or KLDS1.0391 (Jia et al., 2017; Zhang et al., 2018).

**Figure 6:**
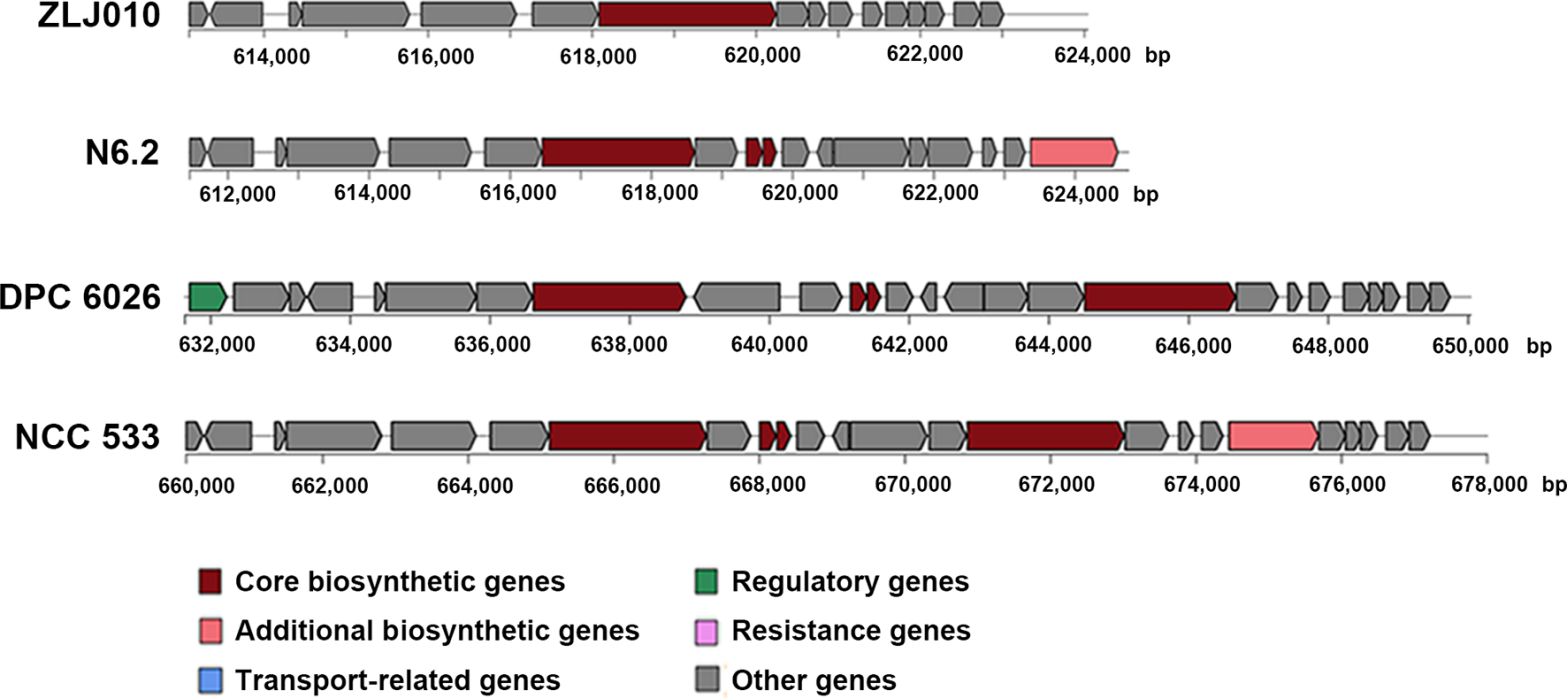
The gene clusters encoding the secondary metabolic enzymes that produce bacteriocin in the genome of *Lactobacillus johnsonii* ZLJ010, N6.2, DPC6026, and NCC 533.

The ZLJ010 genome encoded a limited number of copies of a secondary metabolic enzyme, a nonribosomal peptide synthetase (NRPS) (**Figure 6** and **Table S10**). This NRPS, encoded by a 12.2-kb gene cluster containing 16 genes (gene0589–0604), was predicted to produce bacteriocin, which exhibited 44% similarity to the bacteriocin gassericin T. The ZLJ010 genome harbored fewer copies than the *L. johnsonii* NCC 533, DPC 6026, and N6.2 strains of the core biosynthetic gene (gene0595) encoding the bacteriocin cleavage/export ABC transporter and also lacked regulatory or resistance genes.

*L. johnsonii* is a changing and versatile species. The potential genomic diversity and rearrangements within the *L. johnsonii* species are largely driven by the selective pressure of the differing environment. The results of the present study confirm the role of *L. johnsonii* ZLJ010 as the genetic basis for its survival and probiotic activities and also yield potential candidate genes that may help expand the further application of *L. johnsonii* ZLJ010 in humans and animals.

## Author Contributions

WZ and HJ conceived and designed the experiments. WZ, DZ, HL, SW, and YW performed the ZLJ010 cultivation and DNA extraction. WZ and JW performed the genome analysis. WZ and HJ prepared the manuscript.

## Funding

This work was supported financially by the National Key R&D Program of China (Project no. 2017YFD0502200), National Natural Science Foundation of China (Project no. 31702303), the Specific Public Welfare Program of BAAFS (Project No. XMS201803), the Special Program on Science and Technology Innovation Capacity Building of BAAFS (grant no. KJCX201914), and Beijing Innovation Consortium of Agriculture Research System (grant no. BAIC02-2019).

## Conflict of Interest Statement

The authors declare that the research was conducted in the absence of any commercial or financial relationships that could be construed as a potential conflict of interest.
